# Accurate Extraction of the Self-Rotational Speed for Cells in an Electrokinetics Force Field by an Image Matching Algorithm

**DOI:** 10.3390/mi8090282

**Published:** 2017-09-18

**Authors:** Xieliu Yang, Xihui Niu, Zhu Liu, Yuliang Zhao, Guanglie Zhang, Wenfeng Liang, Wen Jung Li

**Affiliations:** 1 School of Mechanical Engineering, Shenyang Jianzhu University, Shenyang 110168, China; yang.xieliu@sjzu.edu.cn (X.Y.); niuxihui@foxmail.com (X.N.); 2 State Key Laboratory of Robotics, Shenyang Institute of Automation, Chinese Academy of Sciences, Shenyang 110016, China; liuzhu@sia.cn; 3 Department of Mechanical and Biomedical Engineering, City University of Hong Kong, Hong Kong 999077, China; zhaoyuliang.cityu@gmail.com; 4 Institute of Advanced and Intelligent Sensing Systems, Shenzhen Academy of Robotics, Shenzhen 518057, China; guanglie.zhang@gmail.com

**Keywords:** cellular behaviors, electrokinetics field, image-tracking, self-rotation, template-matching

## Abstract

We present an image-matching-based automated algorithm capable of accurately determining the self-rotational speed of cancer cells in an optically-induced electrokinetics-based microfluidic chip. To automatically track a specific cell in a video featuring more than one cell, a background subtraction technique was used. To determine the rotational speeds of cells, a reference frame was automatically selected and curve fitting was performed to improve the stability and accuracy. Results show that the algorithm was able to accurately calculate the self-rotational speeds of cells up to ~150 rpm. In addition, the algorithm could be used to determine the motion trajectories of the cells. Potential applications for the developed algorithm include the differentiation of cell morphology and characterization of cell electrical properties.

## 1. Introduction

The study of cellular behaviors is of significant importance for exploring and elucidating the intrinsic properties of cells, such as their electrophysiological [1], biomechanical [2], and dielectric properties [3]. In general, these intrinsic properties correlate with human disease, so understanding cellular properties can improve our understanding of the effects of cancer treatments [4]. A series of studies on this topic have already been reported. For example, the stiffness of cancer cells with the highest migration and invasion potential is only one-sixth that of cells with the lowest migration and invasion potential based on rapid characterization of cellular biomechanical properties [5]. This observation clearly demonstrates the contribution of the mechanical properties of cells to invasion and suggests that pathways affecting mechanical phenotypes can be targeted as a new approach for molecular cancer therapy. Furthermore, by measuring the dielectric parameters of adult stem cell differentiation, it has been shown that dielectric differences can be determined and exploited to perform real-time and label-free monitoring of stem cell differentiation with impedance sensing [6]. Accordingly, substantial efforts have been dedicated to studies of cell behaviors and their corresponding information acquisition. For instance, a microfluidics-based approach can easily determine the mechanical and electrical parameters of cells by investigating mechanical deformation and translation behaviors, respectively [7,8]. That is, microfluidics enables the determination of cellular states and the identification of different types of cells. However, it is not easy to automate this method because it depends on custom-designed microstructures. A promising alternative is the alternating current (AC) electrokinetics-based technique, which mainly involves dielectrophoresis (DEP) [9,10] and electro-rotation (EROT) [11,12], and can be used to determine the dielectric parameters of cells by describing cellular translational/rotational motions under an externally applied AC bias potential in a non-invasive and label-free manner. The effect of EROT on cells can be broadly classified into two categories based on the different electric field conditions: (1) the cells rotating within a rotational AC electric field due to a phase difference in the AC bias potential between the neighboring electrodes; and (2) certain types of cells with specific inherent dielectric properties will also self-rotate in a linearly polarized (i.e., non-rotational) AC electric field. Zimmermann U. et al. reviewed the electro-rotation of multiple cells and single cells in rotational electric field [13], which was a typical form of cell rotation. As an application of this type of rotation in rotating electric field, it was employed to manipulate and characterize human malignant cells by combing with DEP and travelling DEP techniques [14]. The EROT in rotating field was also used to manipulate cells, and results showed the frequency variation of the spin and orbital torques of cells in a rotating field [15]; dielectric behavior of cell aggregate was further examined. In addition, it was adopted to characterize dielectric parameters of cells during the cellular permeabilization by combing negative DEP to trap cells of interest [16]. On the other hand, the second type of EROT was also studied. For example, Quincke G. reported the Quincke rotation, meaning the cell can rotate in no rotating field; in this case, the rotational direction of cells cannot be predicted and there exists a definite threshold field [17]. Spin resonance [18], as another type of cell rotation in non-rotational field, was reported by Phol H.A. et al. due to coupling of the induced-dipole and externally applied field. Nevertheless, this phenomenon cannot be confirmed by the present authors. Furthermore, Chuang C.H. et al., introduced the self-rotation of human promyelocytic leukemia cells by seeding nanoparticles into cells, thus acquiring the dielectric parameters of cell [19]. The predominant requirements for this method include a non-uniform electric field and automated control based on computer vision to accurately extract the translational/rotational speeds of the cells. The rotational speeds are usually measured by timing the rotation of an individual cell manually, visualized under video microscopy, using a stopwatch [20,21]. It's tedious and a more efficient method is required. A machine vision algorithm and hardware implementation was developed to automatically measure the rotational speed of mammalian cells [20]. However, it aims at the measurement of a cell’s in-plane rotation and can only deal with the image sequence involving a single cell.

Our group has determined the self-rotation motion of certain cells in this type of electric field [22] generated by the metal-based electrodes and established an algorithm for the characterization of the self-rotational motion of cells, including the in-plane and out-of-plane rotation [23]. The biggest difference between our group and other ones is that individual cells can also rotate in no rotating field with known and controllable rotation direction; there definitely exists no threshold electric field; and there is no need to rotate cells with the help of seeding extra nanoparticles. To overcome the inflexible manner of producing the non-uniform electric field by metal-based electrodes, our group has also used an optically-induced electrokinetics (OEK) chip to explore translational motion and self-rotational motion [24,25,26]. Mostly importantly, we have verified experimentally and theoretically that the individual cells can generate the self-rotational motion in this kind of optically-induced non-uniform and non-rotational field [26,27]. In our previous study, we have discussed the optical power required for OEK [28] and elucidated the temperature increased due to the incident light [29], and results demonstrated that the optical power was five orders lower than the optical tweezer [28] and the increased temperature arising from the optical power could be neglected [29]. Hence, this method is a much lower power and non-invasive, which was widely employed into manipulation and separation of cells [24,25,26] and label-free and non-destructive isolation of circulating tumor cells in a larger population [30]. However, the algorithm developed in [23] first computes the translational motion vector of the cell by minimizing the sum of the absolute difference between a rotatable template patch in the reference frame and the target patch in the current frame, and, after compensation for the translational motion, the frame indexes corresponding to the local maximum values of the correlation coefficients between the template patch and the patches at the same location in the sequence frames are used to estimate the self-rotational speed of the cell. That algorithm only roughly extracts the cells’ self-rotational speed and requires human intervention. To further improve the accuracy of determining a cell’s rotation and automate the entire computational process, we have recently developed a novel matching-based detection algorithm to overcome the limitations of the prior method. Compared with the previous algorithm [23], the extraction process of the self-rotational speed is simplified and optimized. In addition, three key techniques are employed in the new algorithm, enabling the following tasks to be performed: (1) a background subtraction technique enabled an automatic tracking of the cell of interest in a video involving many cells undergoing different motions; therefore, it is no longer necessary to select the cell of interest in each frame of the video manually, which greatly increased the efficiency of analysis; (2) automatically select an image distinct from its adjacent images as the reference image for matching, eliminating the need to observe the videos repeatedly and select the reference image manually, and thus reducing the dependence of the results on human intervention and achieving greater stability; (3) a curve-fitting technique enabled a more accurate determination of the time needed for one revolution of the cell of interest, thus increasing the accuracy of the obtained self-rotational speed.

In this paper, we will show the self-rotational behavior of a cell of interest can be accurately and automatically characterized from a video of many cells undergoing different motions by using the new algorithm. In addition, the motion trajectory of a cell of interest can be also determined by the new algorithm, which enables a comprehensive analysis of cellular behaviors. Hence, this algorithm is highly beneficial to the development of new label-free biomarkers for characterization of cellular state, discrimination of different kinds of cancer cells in heterogeneous populations, and many other biomedical and bioengineering related applications.

## 2. Theory

As a whole, nearly all of the applied voltage dropped across the hydrogenated amorphous silicon (a-Si:H) layer when the OEK chip was not illuminated by visible light, due to its inherent lower conductivity. Instead, when an optical pattern from a commercial digital projector was projected onto the surface of the a-Si:H, the electron-hole pairs were excited and enhanced by the migration of electrons from the valence band to the conduction band of the a-Si:H layer, thus locally increasing the conductivity of the a-Si:H via the photoconductive effect. Then, the electric field across the liquid chamber dramatically increased above the locally illuminated a-Si:H area because most of the applied voltage was substantially shifted to the liquid chamber. Accordingly, a nonuniform electric field could be created in the liquid chamber and then any suspended particles at locations in the vicinity of this optically-induced nonuniform electric field would experience a force though an interaction between the electrically polarized dipole moments of both of the particles and the liquid solution, known as the DEP function, which is defined as the “optically induced dielectrophoresis (ODEP) force” in this OEK chip. Unlike conventional DEP chips, no metal electrodes were required to create the non-uniform electric field. The ODEP force could be either positive or negative under specific conditions, meaning that the particles could be either attracted to or repelled from the illuminated areas due to the positive or negative DEP force, respectively. The time-averaged DEP force acting on a spherical nanoparticle is defined as [31]

(1) 〈F→DEP〉=2πR3εmRe[K(ω)]∇|E→rms|2

where *R* is the cell radius; *ε*_m_ denotes the permittivity of the liquid medium; *E*_rms_ is the root-mean-square (rms) value of the electric field; *ω* is the angular frequency with an expression of *ω* = 2π*f*, where *f* is the applied voltage frequency across the liquid medium; and Re[*K*(*ω*)] is the real part of the Clausius–Mossotti (CM) factor representing the direction of the DEP force. For the cell, the expression of Re[*K*(*ω*)] is typically expressed as [31]

(2) Re[K(ω)]=Re[−ω2(τ1τ2−τcτ2′)+jω(τ2′−τ1−τ2)−1ω2(τcτ2′+2τ1τ2)−jω(τ2′+2τ1+τ2)−2]

where *τ*_c_ = *ε*_c_*/σ*_c_, *τ*_1_ = *ε*_m_*/σ*_m_, *τ*_2_ = *C*_mem_*R/σ*_c,_ *τ*_2_*’= C*_mem_*R/σ*_m_; the subscripts c, mem and m represent the cell cytoplasm, cell membrane, and liquid medium, respectively; *C*_mem_ is the capacitance of the cell membrane; *ε* and *σ* are the permittivity and conductivity, respectively. If a cell is attracted to a region of higher electric field, it is said to be “pulled” by a positive DEP force, whereas if the particle is pushed toward a region of lower electric field, it is said to be “pushed” by a negative DEP force.

However, in our previous study [24,26], we have reported that the ODEP can only produce the translational motion of cells and cannot result in the self-rotation of some specific types of cells. In fact, the rotation theory and mechanism in a rotational AC electric field have been clearly defined and understood by researchers; nevertheless, cells rotating in a linearly polarized AC electric field (i.e., non-rotational property) are rarely observed, and this phenomenon has been frequently questioned and argued since it was firstly reported [18,32]. Turcu published a theoretical analysis to explain the reason why certain types of cells will self-rotate in a non-rotational AC electric field along an axis perpendicular to the electric field lines; a possible confirmation that these phenomena may exist under specific conditions [33]. To the best of our knowledge, the theory on this topic from Turcu can well explain the self-rotation motion of some specific cells in non-rotational electric field. In addition, our group described the rotational behavior of pigmented cells with different intrinsic melanin content in a linearly polarized AC electric field; we also experimentally confirmed the rotation phenomenon by seeding foreign particles into cells [22]. Prior to seeding, these cells did not originally self-rotate in the presence of an externally applied non-rotational AC electric field. In general, the self-rotation equation is defined as [33]

(3) $$T=\frac{9}{4}V\varepsilon_{1}E_{0}^{2}\frac{\varepsilon_{r}-\sigma_{r}}{\left(\varepsilon_{r}+2\right)\left(\sigma_{r}+2\right)}\left(\frac{X+X_{0}}{{\left(X+X_{0}\right)}^{2}+1}+\frac{X-X_{0}}{{\left(X-X_{0}\right)}^{2}+1}\right)$$

where *V* is the volume of cell. The other symbols are as follows

(4) $$\{\begin{array}{l} \varepsilon_{r}=\frac{\varepsilon_{p}}{\varepsilon_{m}}\\ \sigma_{r}=\frac{\sigma_{p}}{\sigma_{m}}\\ \omega_{0}\tau =X_{0}\\ \omega \tau =X\\ \tau =\frac{\varepsilon_{p}+2\varepsilon_{m}}{\sigma_{p}+2\sigma_{m}} \end{array}$$

where *ω*_0_ is the angular frequency of cellular motion. From the above Equations, we can easily see that the intrinsic information of cells can be obtained when self-rotation motion versus the frequency is extracted experimentally.

## 3. Materials and Methods

### 3.1. Experimental Setup and Working Principle

**Figure 1:**
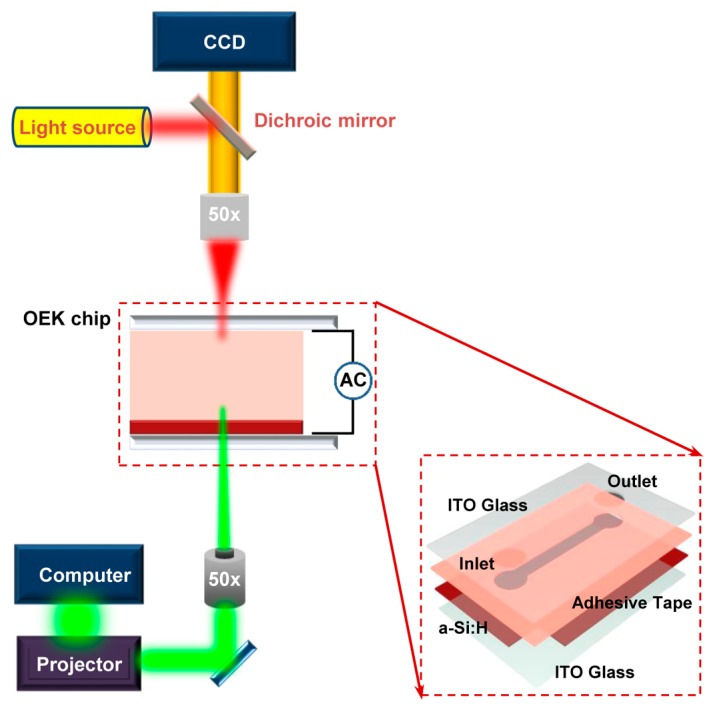
Schematic illustration of the experimental setup for the optically-induced electrokinetics (OEK) platform. The inset is the exploded-view illustration of the OEK chip.

Figure 1 schematically illustrates the experimental setup for the OEK-based microfluidics platform. We have given a detailed introduction on this system in our previous work [26]. The cellular motion was observed and recorded by a charge-coupled-device (CCD) camera (DH-SV1411FC, Beijing Daheng Image Vision Co., Ltd., Beijing, China) fixed on a microscope (Zoom 160, OPTEM, Qioptiq, St Asaph, UK). The OEK chip, as depicted in the inset of Figure 1, was composed of four layers: a top glass substrate with a transparent thin-film of conductive indium tin oxide (ITO); a hydrogenated amorphous silicon (a-Si:H) thin film deposited onto the bottom ITO glass substrate; and a fabricated adhesive tape with the custom-designed microchannel connecting the top ITO glass layer and the a-Si:H layer.

When not illuminated, the a-Si:H layer behaves as an insulator due to its inherent lower conductivity. When a designed digital light pattern is projected onto any desired area of the a-Si:H layer, the conductivity of the illuminated area is sharply increased. Hence, the externally applied AC potential is shifted to the liquid chamber. The electric field across the liquid chamber dramatically increases above the locally illuminated a-Si:H area. Thus, a non-uniform electric field can be produced in the liquid chamber. We previously demonstrated that this type of optically induced non-uniform electric field is also non-rotational [26].

### 3.2. Cell Preparation

A Raji cell line was purchased from the cell bank of the Chinese Academy of Sciences, Shanghai, China. The Raji cells were cultured in Roswell Park Memorial Institute (RPMI-1640, Thermo Fisher Scientific, Bridgewater, NJ, USA) culture medium supplemented with 10% (*v/v*) fetal calf serum, 1% penicillin (*v/v*) (100 U/mL), and 1% streptomycin (*v/v*) (100 μg/mL) at 37 °C in an incubator with a humidified atmosphere of 5% CO_2_. The diameter of the Raji cells was ~12 μm.

Before each experiment, 1 mL of Raji cell suspension was centrifuged at 1000 rpm for 5 min at 4 °C with the supernatant discarded. The collected Raji cells were resuspended into 1 mL of RPMI-1640 culture medium and centrifuged again using the same parameters to remove the residual culture medium. Then, the remaining Raji cells were resuspended into 1 mL of isotonic solution for further experiments. Herein, the isotonic solution used in our experiments consisted of 8.5% (*w/v*) sucrose, 0.3% (*w/v*) glucose, and 0.5% (*w/v*) bovine serum albumin (BSA) in deionized water, which was the optimum solution capable of effectively treating with the cell adhesion on the substrates described in our previously published paper [26]. The aim of adding BSA was to decrease the affinity force between the cells and the a-Si:H substrate of the OEK chip. In this study, we only focused onto the single cell rotation, and hence during a series of experiments performed in this manuscript, this adhesion phenomenon would not affect the experimental results. The conductivity of the isotonic solution was measured to be 1.5 × 10^−2^ S/m using a conductivity meter (Cond 3110, VWR International, Radnor, PA, USA). After the cellular suspensions were prepared, cells counts were performed using a commercial hemocytometer (Shanghai Qiujing Co., Ltd., Shanghai, China) to keep the cell concentration of 1 × 10^5^ cells/mL constant during the experiments.

### 3.3. Self-Rotational Speed Extraction

In general, there were significant differences in images acquired at different rotational angles when a cell was self-rotating. Therefore, a local rectangular region covering the cell of interest in a reference image was used as the matching template when attempting to find the same rectangular regions in the image sequence of the video by calculating and comparing the correlation coefficients. The time interval between two continuous images containing the same rectangular region was considered the time required for the cell of interest to make a complete revolution; thus, the self-rotational speed was available.

Three key concepts are employed in this algorithm to make the speed extraction automatic, accurate, and reliable. (1) To achieve automated tracking of a cell, the cell is manually selected in the first frame of the video. It is then automatically tracked in the subsequent frames using the background subtraction technique. (2) A reference frame that is quite distinct from its adjacent frames is automatically selected according to the correlation coefficients between adjacent frames. (3) A parabolic curve is fitted for each peak of the maximum correlation coefficient sequence, which is calculated based on the maximum correlation coefficients between each frame of the video and the reference frame.

The detailed procedure is explained below. Note that all original (RGB) images captured by the CCD camera were first converted to grayscale before the following processing steps.

#### 3.3.1. Selection of a Cell of Interest in the First Frame

**Figure 2:**
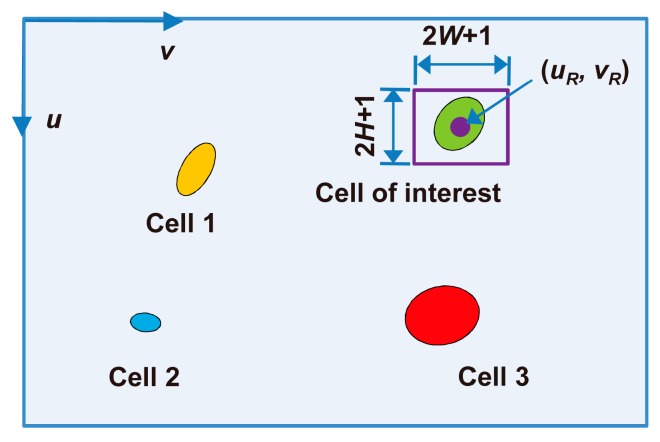
Selection of the cell of interest in the first frame. The cell of interest was manually selected with a rectangle, whose size should be as small as possible under the premise of covering the cell of interest.

The video frames usually contained multiple cells. To analyze the behavior of the cell of interest, the cell was manually selected with a rectangle in the first frame. The center, width, and height of the rectangle were denoted by (*u_R_*, *v_R_*), 2*W* + 1 and 2*H* + 1, respectively (see Figure 2). The cell of interest was covered by the rectangle, but the size of the rectangle was minimized to reduce the impact of non-cell regions on the following correlation calculations.

#### 3.3.2. Tracking the Cell of Interest

**Figure 3:**
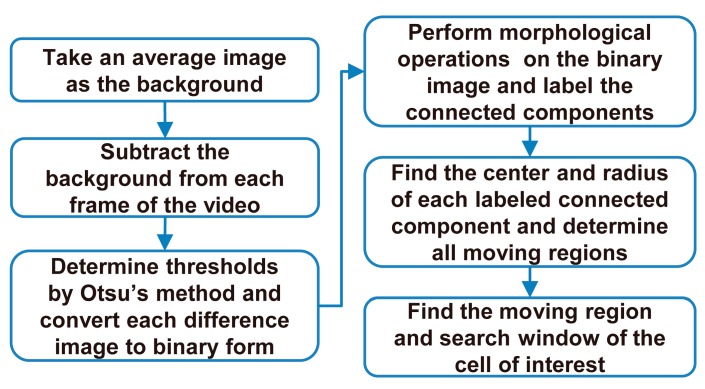
Automated tracking of the cell of interest in the video.

The moving regions of all non-stationary cells were located in each frame of the video by background subtraction, and the moving region of the cell of interest was determined. A rectangular region covering the cell of interest, called the search window, was defined and specified in each frame to enable tracking of the cell of interest. Figure 3 shows the flow of cell tracking.

The background image was obtained using the formula

(5) $$f_{B}=\frac{1}{N}\sum_{i=1}^{N}f_{i}$$

where *f*_B_ represents the background image, *f_i_* denotes the *i*th frame of the video and *N* is the number of frames used for calculating the background image. To guarantee the reliability of the background image, *N* must be greater than the number of frames captured in a self-rotational cycle.

The difference image ∆*f_i_* was obtained by subtracting the background image from each frame of the video

(6) $$\Delta f_{i}=\left|f_{i}-f_{B}\right|$$

Otsu’s method [34], a classical threshold selection method that chooses a threshold to maximize the between-class variance, was then used to determine a threshold *level*, and the grayscale difference image was converted into binary form:

(7) $$\Delta f_{i}(u,v)=\{\begin{array}{c} 1\;\;\;\;\;\mathrm{if}\;\Delta f_{i}(u,v)>level\\ 0\;\;\;\;\;\mathrm{if}\;\Delta f_{i}(u,v)\leq level \end{array}$$

**Figure 4:**
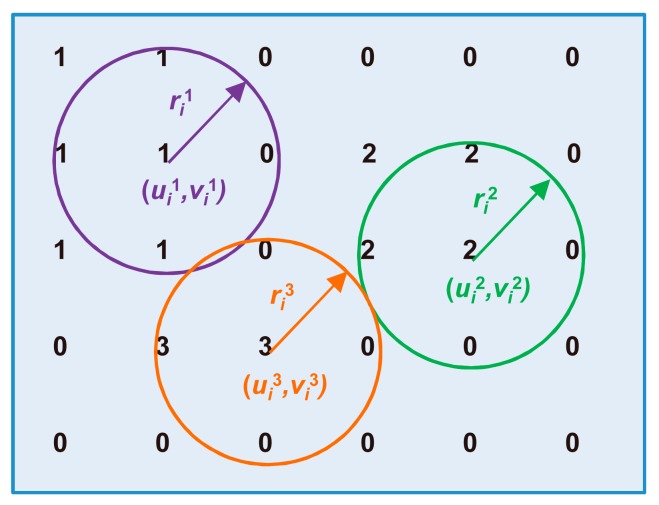
Illustration of the center and radius of each labeled connected component.

A flat, disk-shaped structural element with radius *R* (the structural elements consisted of pixels whose centers were no greater than *R* from the origin) was used to perform morphological opening on the binary image ∆*f_i_* to remove smaller noises, and morphological closing was then used to connect adjacent components. The connected components in ∆*f_i_* were labeled afterward. The center and radius of each connected component, denoted by (*u_i_^q^*, *v_i_^q^*) and *r_i_^q^* (illustrated by Figure 4), respectively, were calculated as follows:

(8) $$\{\begin{array}{l} u_{i}^{q}=\mathrm{Ro}(\frac{1}{M_{i}^{q}}\sum_{m,n}u_{imn}^{q})\\ v_{i}^{q}=\mathrm{Ro}(\frac{1}{M_{i}^{q}}\sum_{m,n}v_{imn}^{q})\\ r_{i}^{q}=\mathrm{Ro}\{\underset{m,n}{\max}{\left[{\left(u_{imn}^{q}-\sum_{m,n}\frac{u_{imn}^{q}}{M_{i}^{q}}\right)}^{2}+{\left(v_{imn}^{q}-\sum_{m,n}\frac{v_{imn}^{q}}{M_{i}^{q}}\right)}^{2}\right]}^{1/2}\} \end{array}$$

where the subscript *i* indicates the image number, the superscript *q* denotes the connected component number, $\left(u_{imn}^{q},\;v_{imn}^{q}\right)$ represents the coordinate of a pixel in the component, *M_i_^q^* denotes the number of pixels in the component, and Ro (·) and max (∙) represent the rounding and maximum searching functions, respectively.

**Figure 5:**
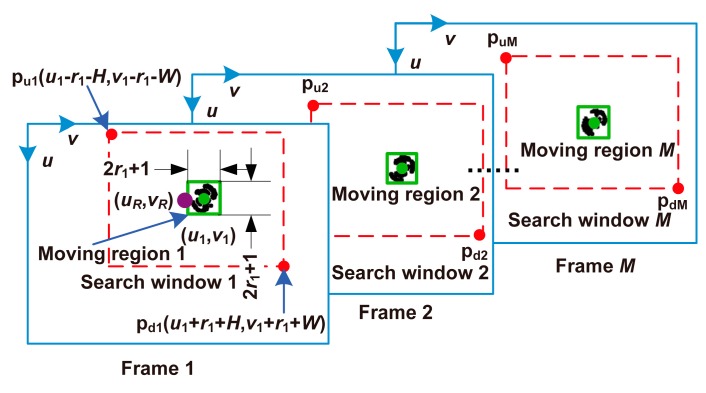
Moving regions and search windows of the cell of interest. (*u_R_*, *v_R_*), 2*W* + 1 and 2*H* + 1 are the center, width, and height of the rectangular region selected in the first frame. *M* denotes the number of frames in the video.

In the first frame of the video, the center of the connected component closest to (*u_R_*, *v_R_*) (the center of the manually selected rectangular region mentioned above) was taken as the center of the moving region of the cell of interest in the first frame, denoted by (*u*_1_, *v*_1_), and the radius of this connected component was denoted by *r*_1_. The rectangular region with its center at (*u*_1_, *v*_1_) had both width and height of 2*r*_1_ + 1 and was regarded as the moving region of the cell of interest in the first frame. To simplify the expression, the rectangular region with center (*u*_1_, *v*_1_) has a width and height of *w* and *h* pixels, respectively, and is referred to Rect [(*u*_1_, *v*_1_), *w*, *h*] in the following sections. By searching the center of the connected component in the *i*th frame, which was closest to (*u_i_*_−1_, *v_i_*_−1_), the moving region of the cell of interest in the *i*th frame, i.e., Rect [(*u_i_*, *v_i_*), 2*r_i_* + 1, 2*r_i_* + 1], was identified. Thus, the moving region of the cell of interest in each frame of the video was identified. Rect [(*u_i_*, *v_i_*), 2*r_i_* + 2*W* + 1, 2*r_i_* + 2*H* + 1] was taken as the search window of the cell of interest. Figure 5 illustrates the tracking process.

#### 3.3.3. Determination of the Reference Frame

**Figure 6:**
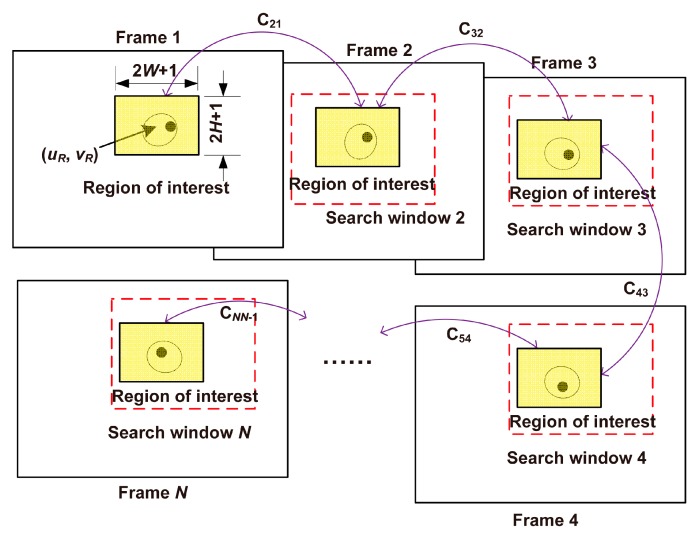
Schematic illustration of the selection of the reference frame. The rectangular regions surrounded by the red dashed lines show the search windows which were determined in Section 3.3.2. The yellow rectangular region in each frame shows the region of interest, whose center is denoted by (*u_Ri_*, *v_Ri_*), where *i* ∈ [1, *N*].

The correlation coefficient between two image regions with the same size shows the similarity of the two regions. In this paper, the normalized correlation coefficient was used, defined by Equation (9). The greater the correlation coefficient is, the more similar the two regions are. To make the measurement of the self-rotational speed more accurate, a frame in which the cell of interest looks significantly different from its adjacent images should be selected as the reference frame. That means a reference frame should have small correlation coefficient with its former frame as well as its latter frame about the local image region covering the cell of interest. To find such a reference frame, the rectangular region previously manually selected in the first frame (i.e., Rect [(*u_R_*, *v_R_*), 2*W* + 1, 2*H* + 1], see Section 3.3.1) was taken as the region of interest in the first frame. For each of the other frames of the video, the region of interest is defined as the region with maximum correlation coefficient with the region of interest in the former frame. Replacing the subscript *j* with *i* − 1 for Equation (11), the center (*u_Ri_*, *v_Ri_*) of the region of interest in the *i*th frame and the maximum correlation coefficient *C_ii-_*_1_ between the *i*th and (*i* − 1)th frame can be calculated according to Equation (11) ( illustrated by Figure 6), where *i* ∈ [2, *N*]. Thus, the maximum correlation coefficient sequence [*C*_21_, *C*_32_, …, *C_NN_*_−1_] can be obtained. Sort the maximum correlation coefficient sequences [*C*_21_, *C*_32_, *C*_43_, …, *C_N_*_−1*N*−2_] and [*C*_32_, *C*_43_, *C*_54_, …, *C_NN_*_−1_] in ascending order, respectively. The sorted sequences are denoted by *FS* and *BS*. The *i*th frame corresponding to the minimum sum of the ordinal number of *C_ii_*_−1_ in *FS* and the ordinal number of *C_i_*_+1*i*_ in *BS* is selected as the reference frame (see supplementary material Figure S1 for the pseudo code). The reference frame was denoted by *f_T_*, and the corresponding Rect [(*u_RT_*, *v_RT_*), 2*W* + 1, 2*H* + 1] was the region of interest in the reference frame.

(9) $$R_{ij}(u_{p},v_{p})=\frac{\sum_{n=-W}^{W}\sum_{m=-H}^{H}\left[f_{j}(u_{Rj}+m,v_{Rj}+n)-\bar{f_{j}}(u_{Rj},v_{Rj})\right]\left[f_{i}(u_{p}+m,v_{p}+n)-\bar{f_{i}}(u_{p},v_{p})\right]}{{\left\{\sum_{n=-W}^{W}\sum_{m=-H}^{H}{\left[f_{j}(u_{Rj}+m,v_{Rj}+n)-\bar{f_{j}}(u_{Rj},v_{Rj})\right]}^{2}\sum_{n=-W}^{W}\sum_{m=-H}^{H}{\left[f_{i}(u_{p}+m,v_{p}+n)-\bar{f_{i}}(u_{p},v_{p})\right]}^{2}\right\}}^{1/2}}$$

where $\bar{f_{j}}$ (*u_Rj_*, *v_Rj_*) represents the mean gray of Rect [(*u_Rj_*, *v_Rj_*), 2*W* + 1, 2*H* + 1] in *j*th frame, and $\bar{f_{i}}$ (*u_p_*, *v_p_*) represents the mean gray of Rect [(*u_p_*, *v_p_*), 2*W* + 1, 2*H* + 1] in *i*th frame, which can be calculated according to Equation (10).

(10) $$\bar{f}(u,v)=\frac{1}{(2W+1)(2H+1）}\sum_{n=-W}^{W}\sum_{m=-H}^{H}f(u+m,v+n)$$

(11) $$\left\{ \begin{array}{l} \left(u_{Ri},v_{Ri})=\underset{(u_{p},v_{p})\in S_{i}}{\arg\max}[R_{ij}(u_{p},v_{p})\right]\\ C_{ij}=R_{ij}(u_{Ri},v_{Ri}) \end{array}\right.$$

where S*_i_* represents the moving region of the cell of interest in the *i*th frame (see Section 3.3.2) and *R_ij_* (*u_p_*, *v_p_*) denotes the correlation coefficient between two regions in the *i*th and the *j*th frame, which is defined by Equation (9).

#### 3.3.4. Calculation of the Self-Rotational Speed

The maximum correlation coefficient *C_iT_* between each frame of the video and the reference frame were calculated according to Equation (11) by replacing the subscript *j* with *T*. The corresponding center (*u_Ri_*, *v_Ri_*) of each region of interest was also acquired using Equation (11). The local maxima of the sequence {*C_iT_*} greater than a specified threshold *lbound* were regarded as the peak points of this sequence. The threshold *lbound* was slightly manually adjusted to avoid choosing the wrong peak points. Then, each peak point and the two points on either side were used to calculate a parabolic curve. The time interval between the maximum points of the adjacent parabolic curves was considered the time required by the cell of interest to complete one revolution. Thus, the average self-rotational speed for each revolution was determined by

(12) $$n=\frac{60f_{\mathrm{fps}}}{X_{i}-X_{i-1}}$$

where *f*_fps_ represents the frame rate of the video (in the experiments shown in the following section, *f*_fps_ = 15 fps), *X_i_* and *X_i−_*_1_ denote the frame numbers of the adjacent maximum points, and *n* is the average self-rotational speed (in units of rpm). The motion trajectory of the cell of interest was estimated using (*u_Ri_*, *v_Ri_*), which are corresponding to the peak points of the maximum correlation coefficient sequence.

## 4. Results and Discussions

### 4.1. Self-Rotational Speed of a Raji Cell under Given AC Bias Parameters

**Figure 7:**
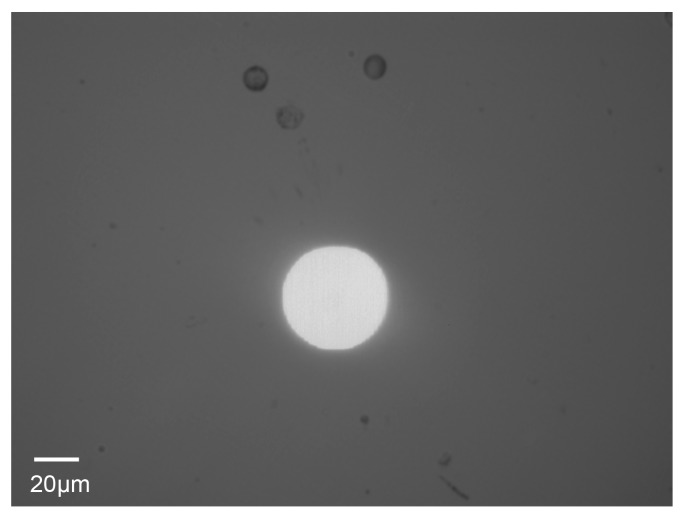
The background image calculated using the first 40 frames.

**Figure 8:**
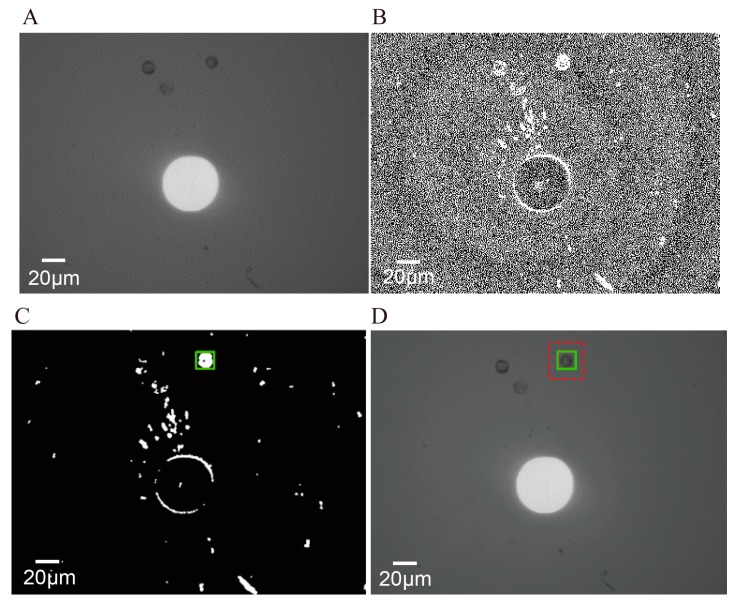
The determined search window of the cell of interest in the 100th frame. (**A**) The 100th frame in grayscale. (**B**) The binary difference image obtained using the threshold determined by Otsu’s method. (**C**) The resulting image when morphological opening and closing operations were applied. The radius *R* of the disk-shaped structural element used here is three pixels. The rectangular region surrounded by green edges is the tracked moving region of the cell of interest. (**D**) The determined search window (the rectangular region surrounded by the red edges, i.e., the dashed lines) of the cell of interest. The green edges (the solid lines) show the tracked moving region in the grayscale image.

**Figure 9:**
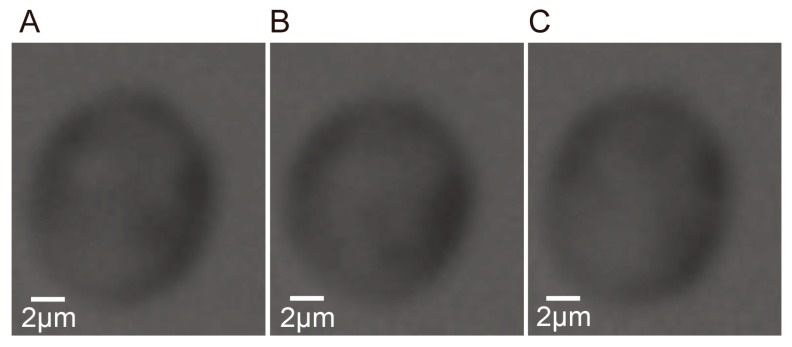
Regions of the cell of interest in the reference frame and adjacent frames. (**A**) The region in the reference frame. (**B**) The region tracked in the previous frame. (**C**) The region tracked in the subsequent frame.

**Figure 10:**
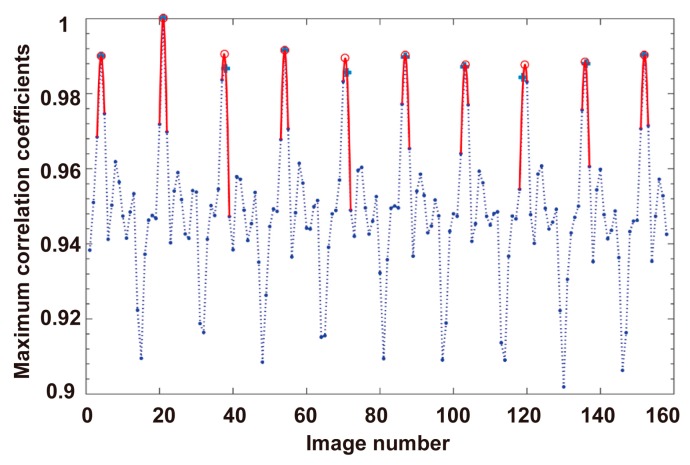
Maximum correlation coefficients between each frame of the video and the reference frame. The blue symbol “+” in shows the peak points of the maximum correlation coefficient sequence. The red symbol “o” denotes the maximum points of the calculated parabolic curves.

**Figure 11:**
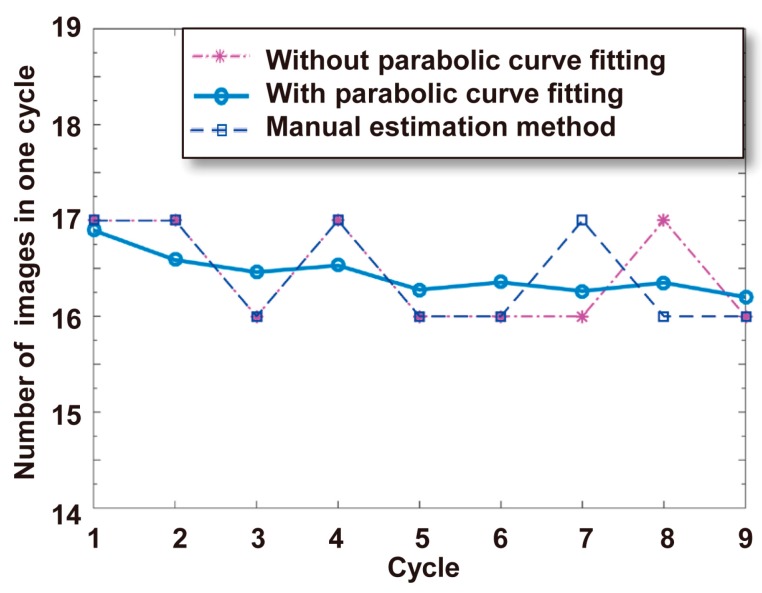
The number of images in each cycle. The data “Without parabolic curve fitting” represent the number of images between two adjacent peak points of the maximum correlation coefficient sequence. The data “With parabolic curve fitting” are the number of images between two maximum points of the adjacent parabolic curves. The data “Manual estimation method” were obtained by observing and comparing the video images frame by frame.

**Figure 12:**
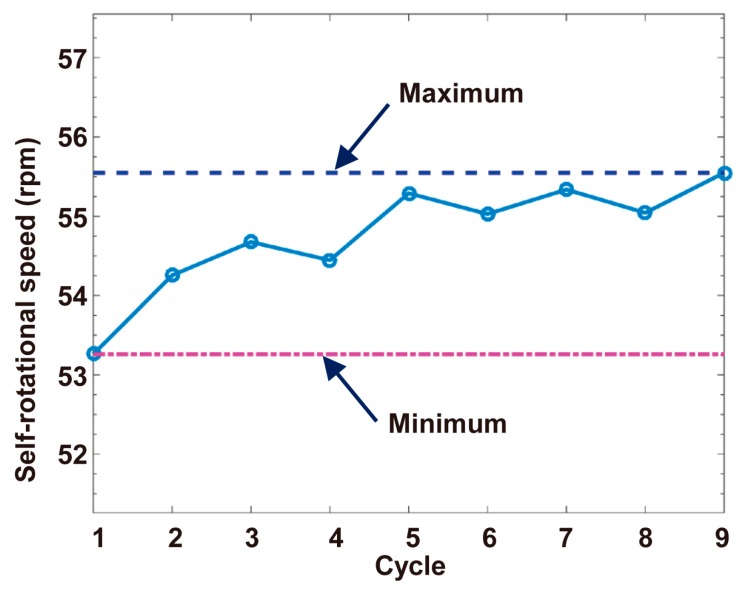
Average self-rotational speed in each cycle.

**Figure 13:**
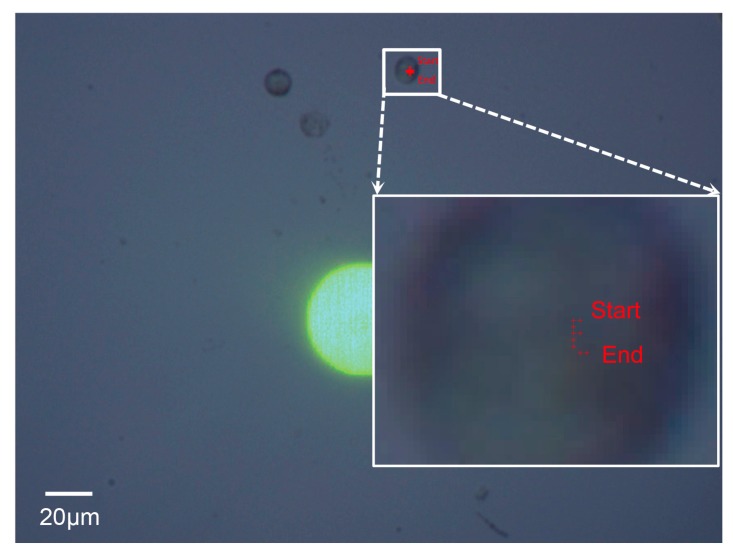
Motion trajectory of the cell of interest.

In this experiment, a Raji cell with a diameter of ~12 μm was observed. The applied AC frequency and bias potential were 75 kHz and 10 V_pp_, respectively. A total of 158 images acquired when the Raji cell was stably self-rotating were analyzed, and the first 40 frames were used to calculate the background and reference frame. The obtained background image is shown in Figure 7. Figure 8 shows the determined search window of the cell of interest in the 100th frame, which was randomly selected to illustrate the general situation. The region of interest tracked in the reference frame is shown in Figure 9. The maximum correlation coefficients between each frame of the video and the reference frame are shown in Figure 10. As shown in Figure 11, the proposed algorithm was highly consistent with the manual estimation method, which supports the accuracy of the proposed algorithm. The standard deviation of the number of images between two adjacent peak points of the maximum correlation coefficient sequence was 0.5 in the nine cycles, and the standard deviation between two maximum points of the adjacent parabolic curves was 0.2, which demonstrates the effectiveness of the curve-fitting method. The average self-rotational speed in each cycle is shown in Figure 12. The speed ranged from 53.3 rpm to 55.5 rpm, and the average self-rotational speed in the nine cycles was 54.76 rpm. The motion trajectory of the cell of interest is shown in Figure 13.

### 4.2. Self-Rotational Speed of Raji Cells under Various AC Bias Parameters

**Figure 14:**
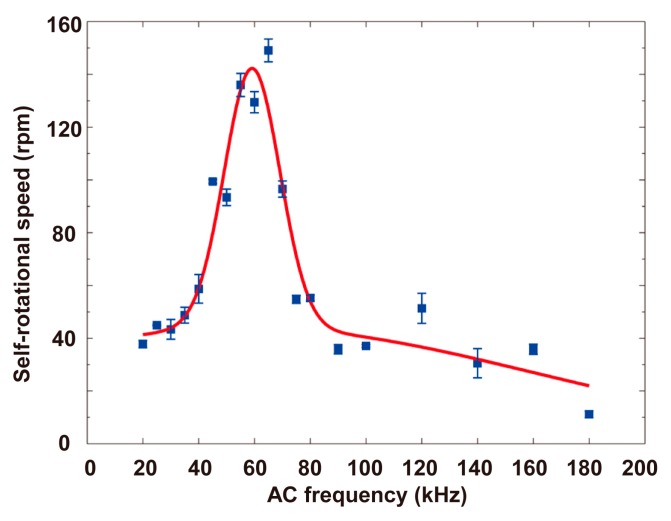
Self-rotational speeds of a Raji cell with different applied AC frequencies. The applied AC bias potential is 10 V_pp_. The measurement points in this figure were obtained for only one Raji cell. At each measurement point, the cell continually rotated for several cycles, and the numbers of rotation cycles are {4, 4, 5, 8, 4, 5, 6, 5, 5, 3, 9, 9, 5, 5, 3, 5, 2, 3, 2} respectively. In the figure, each data point represents a mean value ± maximum deviation. The Raji cell is the same cell observed in the above experiment.

**Figure 15:**
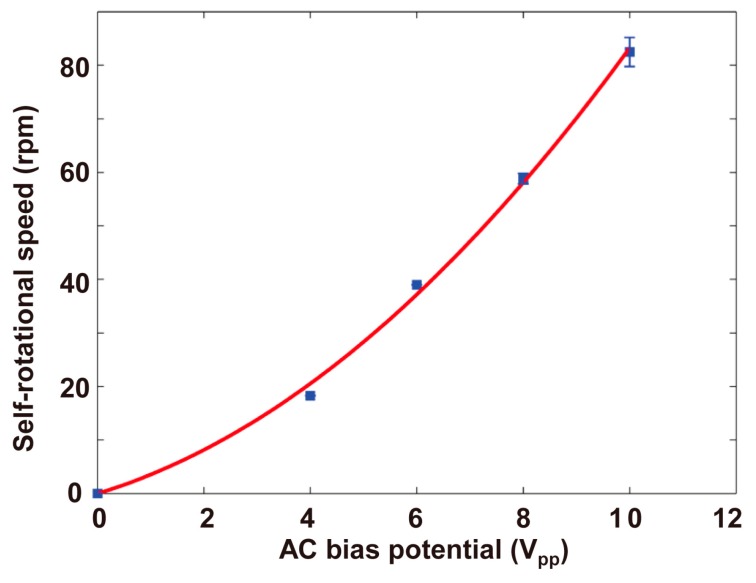
Self-rotational speeds of a Raji cell with different applied AC bias potentials. The applied AC frequency is 60 kHz. The measurement points in this figure were obtained for only one Raji cell. At each measurement point, the cell continually rotated for several cycles, and the numbers of rotation cycles for the applied AC bias potentials {4, 6 8 10} V_pp_ are {1, 1, 3, 5} respectively. In the figure, each data point represents a mean value ± maximum deviation. The diameter of the Raji cell is ~12 μm.

To characterize the self-rotational speeds of Raji cells under different AC bias parameters, the applied AC frequency and bias potential were changed. Figure 14 shows the self-rotational speeds of a Raji cell under different AC frequencies at a constant AC bias potential of 10 V_pp_. The Raji cell self-rotated at the applied frequencies ranging from 20 kHz to 180 kHz, and the maximum self-rotational speed was 149.0 ± 4.3 rpm at a frequency of 65 kHz. Figure 15 shows the self-rotational speed of another Raji cell as a function of applied AC bias potentials from 0 V_pp_ to 10 V_pp_ at a constant frequency of 60 kHz. In the experiments, the self-rotational speed of the Raji cells ranged from 11.1 rpm to 149.0 rpm, without considering the stationary state, and the proposed algorithm was successfully applied under all the conditions.

### 4.3. Discussions

The proposed algorithm was successfully used to analyze the self-rotational and translational motions of cells with non-uniform structure. In all experiments conducted, the cell of interest was correctly tracked. No human intervention was required for the self-rotational speed extraction algorithm, except for the assignment of the cell of interest in the first frame. The algorithm exhibited good robustness and accuracy even if the cell of interest exhibited non-uniform self-rotation. The use of the algorithm for other types of cells and for a wider range of speeds will be investigated in future studies. It is envisioned that the imaging modalities proposed here can be used in conjunction with other microfluidic rotational techniques, such as the structure based [35,36,37,38,39], the acoustic based [40,41,42,43], or optical based [44,45,46] cell rotation techniques. The calculation of the self-rotational speed is theoretically irrelevant to the speed of translational motion, but a high translational speed may make the tracing of the cell of interest difficult. In this case, a high frame rate is helpful. The main limitation of this algorithm is that it can only handle cells whose images are different at different self-rotational angles, but this is the general situation for flat and spherical cells.

## 5. Conclusions

In this study, a fast, accurate, and automated matching-based algorithm was developed to extract the self-rotational and translational motions of cancer cells in an OEK chip. Once a cell of interest was identified, the cell was manually assigned in the first frame, and a background subtraction technique was used to achieve automated tracking of the cell. This process circumvents the problem of analyzing many moving cells in the same recorded video image frames. An image in which the tracked cell looks significantly different from its adjacent images was automatically selected as the reference frame. The correlation coefficients between each frame of the video and the reference frame were calculated and compared to identify the same images of the tracked cell. A curve-fitting technique was used to more accurately calculate the time required for the tracked cell to complete one revolution, leading to sub-frame level accuracy. The proposed algorithm was used to analyze the self-rotational speeds of Raji cells under various AC bias parameters, and the results demonstrate the effectiveness of the algorithm. In summary, the algorithm discussed in this paper can be used to efficiently, reliably, and accurately extract the self-rotational speed of cells in a microfluidic environment. Potential applications for this algorithm include separating different kinds of cancer cells in heterogeneous populations, discriminating normal cells from cancer cells, and characterization of the morphological and electrical properties of cells.

## Supplementary Materials

The following is available online at www.mdpi.com/2072-666X/8/9/282/s1, Figure S1: Pseudo code for choosing the reference frame.
