# Supplementary material for: Accurate Extraction of the Self-Rotational Speed for Cells in an Electrokinetics Force Field by an Image Matching Algorithm
Source: Micromachines (Basel). 2017 Sep 18;8(9):282. doi: 10.3390/mi8090282 (PMC6190232; doi:10.3390/mi8090282)
Supplement: Supplementary file 1 [file micromachines-08-00282-s001.pdf]

Accurate Extraction of the Self-Rotational Speed for Cells in an Electrokinetics Force Field by an Image Matching Algorithm

Xieliu Yang, Xihui Niu, Zhu Liu, Yuliang Zhao, Guanglie Zhang, Wenfeng Liang and Wen Jung Li

```
1.  F ← [MNC_21, MNC_32, …, MNC_{N-1N-2}]
2.  B ← [MNC_32, MNC_43, …, MNC_{NN-1}]
3.  F_s ← F
4.  Set F_index[i] =i , where  i = 1, 2, ……, N-2
5.  for  i ← 1 to N-3
6.        min ← ∞
7.        for  j ← i to N-2
8.            if  F_s[j]<min
9.            then  min←F_s[j]
10.                   minlabel←j
11.            end  if
12.       end  for
13.       exchange F_s[i] ↔ F_s[minlabel]
14.       exchange F_index[i] ↔ F_index[minlabel]
15.   end  for
16.   Do from Line 3 to Line 15 for B getting B_s and B_index
17.   min ← ∞
18.   for  i ← 1 to N-2
19.       for  j ← 1 to N-2
20.           if  B_index[j] = = F_index[i]  && i+j<min
21.           then  min←i+j
22.                 I←i
23.           end  if
24.       end  for
25.   end  for
26.   ReferenceFrameNo=F_index[I] +1
```

Figure S1. Pseudo code for choosing the reference frame.
